# Going for three: what is the role for triplex testing for HSV-1, HSV-2, and VZV?

**DOI:** 10.1128/asmcr.00152-25

**Published:** 2025-11-04

**Authors:** David R. Peaper

**Affiliations:** 1 Department of Laboratory Medicine, Yale University School of Medicine, New Haven, Connecticut, USA; Rush University Medical Center, Chicago, Illinois, USA

## Abstract

Herpes simplex virus-1 (HSV-1), HSV-2, and varicella zoster virus (VZV) are common causes of mucocutaneous lesions. Diagnostic methods have shifted from culture or direct fluorescent antibody testing to nucleic acid amplification tests (NAATs), but testing is (i) not often undertaken or (ii) often specific for either HSV-1/2 or VZV based upon classic clinical findings associated with typical disease. A recent study by B. W. Buchan and P. Pancholi published in *ASM Case Reports* (2:e00112-25, 2025, https://doi.org/10.1128/asmcr.00112-25) highlights the limitations of this approach, revealing a substantial number of cases where provider-ordered testing missed the causative virus. Accurate viral identification is critical in these cases, as missed or misdiagnosis impacts patient management. Treatment regimens, including antiviral dosage and duration, vary among clinical presentations of HSV-1, HSV-2, and VZV. More importantly, infection control protocols differ among these pathogens. Many cases of VZV require airborne and contact isolation in healthcare settings to prevent nosocomial transmission, a precaution not necessary for most HSV-1/2 infections. In some cases, clinical diagnosis may be insufficient for differentiation among HSV-1, HSV-2, and VZV. More studies are needed to help identify use cases where triplex testing is most appropriate, but broader availability and implementation of triplex NAATs have the potential to promote better patient management and infection prevention practices.

## COMMENTARY

Herpes simplex virus-1 (HSV-1), HSV-2, and varicella zoster virus (VZV) are members of *Alphaherpesvirinae* subfamily of the *Herpesviridae* family of DNA viruses (1, 2). They are important causes of disease in both immunocompetent and immunocompromised patients, predominantly causing non-life-threatening mucocutaneous infections, but severe infections of the eye, central nervous system, liver, lungs, or disseminated infections may occur and are associated with severe morbidity and mortality. They establish latency in nerve ganglia, leading to recurrent infections and/or eruption of lesions long after primary infection (3, 4). Seropositivity for these viruses is high, suggesting that many patients are at risk of recurrence. The clinical significance of these viruses is well established, leading to the availability of diagnostic methods, vaccines (for VZV), and antiviral medications.

Traditional viral cell culture can detect HSV-1, HSV-2, and VZV, but with varying sensitivity and time to detection. Rapid viral culture and direct fluorescent antibody (DFA) testing accelerate the time to diagnosis for these pathogens, but these methods are virus specific (5, 6). Culture and DFA have been supplanted by nucleic acid amplification tests (NAAT) due to their sensitivity and turn-around time, but virus-specific testing remains the norm (5–7). There are over 10 Food and Drug Administration (FDA)-authorized duplex tests for HSV-1 and HSV-2, a single FDA-authorized singleplex test for VZV, and only four FDA-authorized assays for triplex testing for HSV-1, HSV-2, and VZV from mucocutaneous lesions (8). Triplexing may not be compatible with some NAAT systems; it is technically feasible with current technology across multiple platforms from multiple vendors.

Differences in HSV-1 and HSV-2 recurrence rates are well known, and the Centers for Disease Control calls for type-specific testing of new onset genital lesions (9, 10). This likely contributed to the development of duplex assays to differentiate these viruses. A potential factor contributing to limited availability of triplexed assays may be the perceived lack of need due to the specificity of clinical presentation, anatomic location, appearance, and epidemiologic risk factors for lesions caused by HSV-1, HSV-2, and VZV. Indeed, laboratory testing is often not undertaken for these viruses, especially herpes zoster or recurrent lesions in patients with a known history of HSV infection, with providers relying on clinical diagnoses to make management decisions, including prescription of antiviral agents (11, 12). Additionally, testing for all three pathogens where there is high clinical suspicion for a single pathogen would increase the cost of testing for both laboratories through the inclusion of additional reagents and controls, as well as patients and payers.

The classic presentation of these lesions is well summarized by Buchan and Pancholi in a recent study published in *ASM Case Reports* (13). Typically, primary infection with HSV-1 or HSV-2 may be asymptomatic, associated with limited lesions at the site of infection, and/or non-specific signs of infection, including fever and malaise. In contrast, primary infection with VZV (i.e., varicella or chickenpox) is associated with multiple lesions at different stages of development over the skin and potentially mucosal sites. With respect to reactivation, HSV-1 and HSV-2 classically cause recurrent orolabial and genital tract lesions, respectively, and VZV reactivation (i.e., herpes zoster or shingles) has a dermatomal distribution, potentially anywhere on the body, but predominantly in a thoracic or cervical distribution (1, 2, 4).

Despite these classic presentations, it is well known that HSV-1 and HSV-2 can cause genital or orolabial infections, respectively, despite their reversed anatomic localization in classic cases (9, 14, 15). Additionally, there are a number of reports of atypical or non-classical presentations of HSV-1, HSV-2, and VZV leading to delayed diagnoses, sub-optimal treatment, and patient exposures (16–18). Thus, clinical diagnosis alone may be insufficient in some cases.

Buchan and Pancholi contribute to this literature by analyzing a large set of samples submitted for HSV-1/HSV-2 and/or VZV testing among adult and pediatric patients (13). They collected relevant demographic and clinical information on lesion swabs submitted for testing and compared provider-ordered testing to the ultimate virus(es) detected following performance of a triplex assay after completion of standard of care testing. Patterns of test ordering largely matched expectations for classic presentation of these viruses, with oral lesions submitted for HSV testing from younger patients, genital lesions submitted for HSV testing from patients between 20 and 40, and cutaneous lesions common across all ages, but predominating in patients over age 60, with most testing requested for VZV. Importantly, they found a number of cases where the provider-ordered test missed the ultimate virus detected. Interestingly, patients aged 40–60 were enriched in the missed diagnosis population with a number of cases clinically suspected of VZV based on provider order ultimately being positive for HSV-1 or HSV-2.

**Fig 1:**
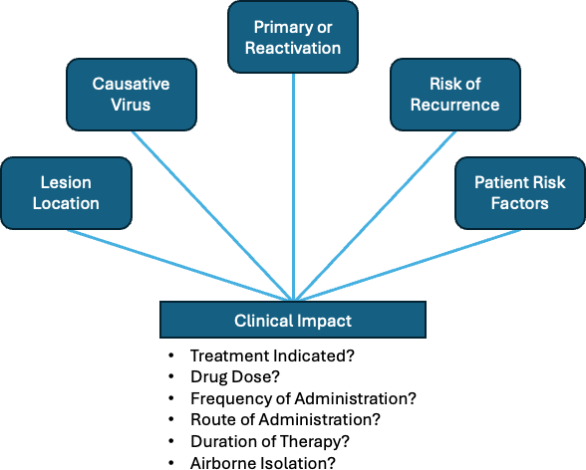
Characteristics of mucocutaneous lesions that may impact the clinical management of patients.

Accurate identification of these viruses can affect patient management, and testing should be undertaken when definitive virus identification could affect patient care (Fig. 1). Guidelines generally call for testing lesions from patients with immunocompromise, but other considerations are as follows: (i) treatment for lesions, including suppressive prophylaxis, varies among HSV-1 and HSV-2 and VZV and (ii) VZV may require a higher level of isolation precautions for admitted patients.

Acyclovir, valacyclovir, and famciclovir are the mainstays of treatment for HSV-1, HSV-2, and VZV, but the dose, frequency of administration, route of administration, and duration of therapy for these medications vary among clinical presentations of primary infection, recurrent infection or reactivation, and chronic suppressive therapy for immunocompetent and immunocompromised patients based on anatomic location, causative virus, and disease severity (9, 19). There is some discordance among commonly used treatment guidelines, but treatment of primary HSV or herpes zoster typically calls for longer durations of higher doses of these medications than treatment of recurrent HSV disease.

Genital outbreaks of HSV-2 have high rates of recurrence, and suppressive therapy is often indicated. In contrast, recurrence of HSV-1 genital infections is less common, and treatment may not be indicated (9, 19, 20). However, VZV activation in the genital area, a described phenomenon also found by Buchan and Pancholi, would necessitate higher treatment doses of medication (21). There would also be important implications for patients and partners for differentiating VZV reactivation from primary HSV-1/2 infection or recurrence of HSV-1/2.

Perhaps more importantly, the mechanism of transmission varies among these pathogens with potential significant implications for isolation precautions in healthcare settings, especially those serving patients with immunocompromise. Patients with lesions caused by HSV-1 or HSV-2 may not need specific isolation precautions (22). In contrast, patients with VZV, either primary infection or recurrence involving more than one dermatome, lesions that cannot be covered, or patients with immunocompromise require airborne and contact isolation (22, 23). The aerosol transmission of VZV poses a high risk to some patients, such as those on oncology wards, as well as at-risk visitors and staff members. Within the hospital, transmission of VZV is well described with potential adverse outcomes (11, 18). Exposed patients and healthcare workers require assessment and potential follow-up (18). There should be a low threshold for triplex testing of lesions on admitted patients to limit the risk of VZV exposures.

The study design precludes precise calculations of the overall rates of potential misdiagnosis or missed diagnosis, but these data argue for wider availability of triplex testing and/or clinical decision support when such testing might be appropriate. A larger, more comprehensive study of HSV-1, HSV-2, and VZV triplex testing may be appropriate to accurately quantify the scope and impact on patient management, including treatment decisions and isolation precautions. In particular, it would be helpful to identify combinations of demographic information and lesion presentation that increase the yield of triplex testing versus clinical diagnosis or targeted testing. Current guidelines for when to test are fairly limited, and these types of studies may help identify more specific indications for testing. Finally, greater commercial availability of triplexed assays would facilitate more widespread testing.
